# Photonuclear production, chemistry, and in vitro evaluation of the theranostic radionuclide ^47^Sc

**DOI:** 10.1186/s13550-019-0515-8

**Published:** 2019-05-16

**Authors:** C. Shaun Loveless, Lauren L. Radford, Samuel J. Ferran, Stacy L. Queern, Matthew R. Shepherd, Suzanne E. Lapi

**Affiliations:** 1 Department of Radiology, University of Alabama at Birmingham, Birmingham, AL 35233 USA; 2 Department of Chemistry, Washington University in St. Louis, St. Louis, MO 63134 USA; 3 Department of Chemistry, University of Alabama at Birmingham, Birmingham, AL 35233 USA; 4 Department of Physics, Indiana University, Bloomington, IN 47405 USA

**Keywords:** ^47^Sc, Photonuclear, Bremsstrahlung, Titanium, Accelerator, eLINAC

## Abstract

**Background:**

In molecular imaging and nuclear medicine, theranostic agents that integrate radionuclide pairs are successfully being used for individualized care, which has led to rapidly growing interest in their continued development. These compounds, which are radiolabeled with one radionuclide for imaging and a chemically identical or similar radionuclide for therapy, may improve patient-specific treatment and outcomes by matching the properties of different radionuclides with a targeting vector for a particular tumor type. One proposed theranostic radionuclide is scandium-47 (^47^Sc, *T*_1/2_ = 3.35 days), which can be used for targeted radiotherapy and may be paired with the positron emitting radionuclides, ^43^Sc (*T*_1/2_ = 3.89 h) and ^44^Sc (*T*_1/2_ = 3.97 h) for imaging. The aim of this study was to investigate the photonuclear production of ^47^Sc via the ^48^Ti(γ,p)^47^Sc reaction using an electron linear accelerator (eLINAC), separation and purification of ^47^Sc, the radiolabeling of somatostatin receptor-targeting peptide DOTATOC with ^47^Sc, and in vitro receptor-mediated binding of [^47^Sc]Sc-DOTATOC in AR42J somatostatin receptor subtype two (SSTR2) expressing rat pancreatic tumor cells.

**Results:**

The rate of ^47^Sc production in a stack of natural titanium foils (*n* = 39) was 8 × 10^7^ Bq/mA·h (*n* = 3). Irradiated target foils were dissolved in 2.0 M H_2_SO_4_ under reflux. After dissolution, trivalent ^47^Sc ions were separated from natural Ti using AG MP-50 cation exchange resin. The recovered ^47^Sc was then purified using CHELEX 100 ion exchange resin. The average decay-corrected two-step ^47^Sc recovery (*n* = 9) was (77 ± 7)%. A radiolabeling yield of > 99.9% of [^47^Sc]Sc-DOTATOC (0.384 mg in 0.3 mL) was achieved using 1.7 MBq of ^47^Sc. Blocking studies using Octreotide illustrated receptor-mediated uptake of [^47^Sc]Sc-DOTATOC in AR42J cells.

**Conclusions:**

^47^Sc can be produced via the ^48^Ti(γ,p)^47^Sc reaction and separated from natural Ti targets with a yield and radiochemical purity suitable for radiolabeling of peptides for in vitro studies. The data in this work supports the potential use of eLINACs for studies of photonuclear production of medical radionuclides and the future development of high-intensity eLINAC facilities capable of producing relevant quantities of carrier-free radionuclides currently inaccessible via routine production pathways or limited due to costly enriched targets.

**Electronic supplementary material:**

The online version of this article (10.1186/s13550-019-0515-8) contains supplementary material, which is available to authorized users.

## Introduction

Theranostic applications in molecular imaging and therapy involve the use of a radionuclide or radionuclide pairs with nuclear, physical, and biological characteristics that allow tailored imaging and therapy in the same patient [1]. This personalized approach to nuclear medicine offers the possibility of matching the properties of different theranostic radionuclides with a targeting vector for distinct tumor types. Important parameters often considered when selecting suitable radionuclides include availability, chemistry, biological properties, half-life, and decay properties. Therefore, the development of new production routes for hard to obtain theranostic radionuclides can provide innovative diagnostic and therapeutic agents for use in personalized nuclear medicine [1–3].

Recently, the development of receptor-targeted radionuclide therapies has highlighted the need for the rapid and complete assessment of their pharmacokinetics [4–6]. To address this need, imaging with a chemically identical diagnostic (labeled with a radionuclide of the same element) affords the truest picture of in vivo distribution for the therapeutic agent [7]. Therefore, the development of matched radionuclide pairs for use in receptor-targeted therapies and imaging is an emerging area of research. Unfortunately, the limited availability of a number of the most promising matched-pair theranostic radionuclides has hindered pre-clinical research and translation of new therapeutic agents to the clinic [7].

The theranostic radionuclide scandium-47 (^47^Sc, *T*_1/2_ = 3.35 days, Eβ^−^_avg_ = 162 keV, Eγ = 159 keV, *I* = 68.4%) has a half-life of 3.35 days and is compatible with the biological half-life of antibodies or peptide-based radiopharmaceuticals [8, 9]. It decays by β^−^ emission with an average β^−^ energy of 162 keV, followed by prompt emission of a 159 keV gamma ray (68.4%) to stable ^47^Ti [10]. Further, ^43^Sc (*T*_1/2_ = 3.89 h, Eβ^+^_avg_ = 476 keV, *I* = 88.1%) and ^44^Sc (*T*_1/2_ = 3.97 h, Eβ^+^_avg_ = 632 keV, *I* = 94.3%) have been proposed as diagnostic radionuclides for positron emission tomography (PET) imaging [11].

Reactor production of ^47^Sc has been investigated using neutron capture on enriched titanium via the ^47^Ti(n,p)^47^Sc reaction (E_n_ > 1 MeV) or neutron capture on enriched calcium ^46^Ca(n,γ)^47^Ca (E_n_ = 0.025 eV), resulting in ^47^Ca (*T*_1/2_ = 4.54 days), which may be used for a ^47^Ca-^47^Sc generator system [12, 13]. One challenge facing development of a ^47^Ca-^47^Sc generator is the natural abundance of ^46^Ca (0.004%). Due to this abundance, it is only available at enrichment levels up to 30% [14]. Accelerator production and measurement of cross sections for the production of ^47^Sc have been investigated using medium to high energy protons (17 < E_p_ < 150 MeV) via the ^nat^Ti(p,2p)^47^Sc, ^48^Ti(p,2p)^47^Sc, ^51^V(p,3pn)^47^Sc, and ^nat^Ca(p,2n)^47^Sc reactions [1, 14, 15]. Cyclotron production via the above methods resulted in co-production of ^46^Sc (*T*_1/2_ = 83.79 days) and ^48^Sc (*T*_1/2_ = 43.67 h), decreasing the radionuclidic purity of ^47^Sc. Production with spallation neutrons was also investigated via the ^47^Ti(n,p)^47^Sc, ^nat^V(n,x)^47^Sc, and ^nat^Ti(n,x)^47^Sc reactions [16]. Based on these data, production routes that afford radionuclidic purity suitable for clinical application require costly enriched targets or access to nuclear reactors with significant high energy neutron flux (E_n_ > 1 MeV). Therefore, the poor availability of ^47^Sc, due to the above requirements, has hampered preclinical investigation and clinical application of the ^47^Sc/^43,44^Sc theranostic matched-pair.

**Fig. 1:**
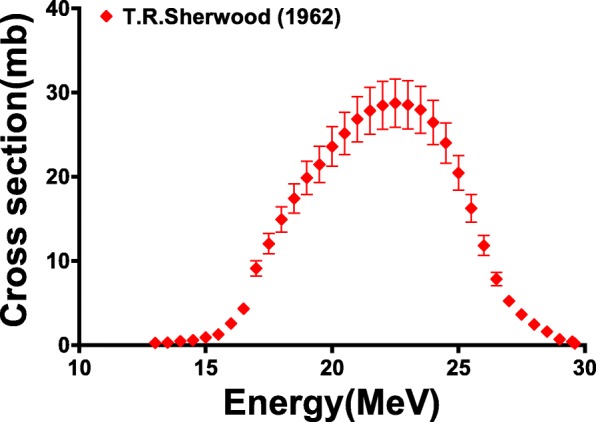
The photonuclear cross section for the sum of the ^nat^Ti(γ,p)^47^Sc and ^nat^Ti(γ,np)^47^Sc nuclear reaction as a function of photon energy

In an effort to address the above challenges, recent work has investigated the use of high-intensity electron linear accelerators (eLINACs) with 20–55 MeV electron beams to produce ^47^Sc via photonuclear reactions on titanium nuclei [17, 18]. The electrons accelerated by these machines emit bremsstrahlung radiation as they pass through matter, resulting in a spectrum of photon energies ranging from zero to the electron energy with a flux that is inversely proportional to the energy of the radiated photon. A small fraction of the bremsstrahlung photons are energetic enough to induce photonuclear reactions such as (γ,n) and (γ,p) for radionuclide production [10, 19]. The flux of bremsstrahlung photons can be enhanced with the use of a radiator or “converter” made from a high Z material, like tungsten. Use of a radiator does not significantly alter the energy distribution of radiated photons, but it enhances the number of radiated photons and hence increases the number that are energetic enough to induce photonuclear reactions. Rotsch et al. (2018) used ^nat^TiO_2_ targets to demonstrate a ^47^Sc production rate at 35 MeV and 40 MeV of 4.25 MBq/g·kW·h and 6.92 MBq/g·kW·h, respectively, using bremsstrahlung photons [20]. Belyshev et al. (2015) showed that the integrated cross section of the ^nat^Ti(γ,p)^47^Sc reaction, using thin titanium foils of natural isotopic composition, was 110 ± 19 mb using a 55 MeV electron beam [18]. T. R. Sherwood and W. E. Turchinetz (1962) measured the cumulative cross section of the ^nat^Ti(γ,p)^47^Sc reaction, yielding a peak cross section of approximately 28 mb at 23 MeV and integrated cross section of 217 ± 32 MeV·mb [21] (Fig. 1).

A high-intensity eLINAC facility capable of photonuclear radionuclide production has many advantages in contrast to reactor or other accelerator facilities. These advantages include reduced volume of radioactive waste, low cost and operation expense, and fewer nuclear reaction channels, leading to potentially higher radiochemical purity of the final product. Previous work on photonuclear production of radionuclides using bremsstrahlung radiation has included ^47^Sc, ^99^Mo for ^99^Mo-^99m^Tc generators, ^67^Cu for the ^67^Cu/^64^Cu theranostic matched-pair, ^225^Ac for targeted alpha therapy, and ^195m^Pt as a theranostic radionuclide [17, 19, 22–24]. Therefore, a number of long-lived theranostic radionuclides or radionuclide pairs, including ^47^Sc, that suffer from lack of availability may be produced by photonuclear reactions at eLINAC facilities.

In this work, the photonuclear production of carrier-free ^47^Sc via the ^nat^Ti(γ,p)^47^Sc reaction using bremsstrahlung radiation generated by an electron beam (22 MeV Varian CLINAC) was investigated. To isolate the produced ^47^Sc from ^nat^Ti target material, a separation method using AG MP-50 cation exchange resin was developed. Following separation, the ^47^Sc was further purified using CHELEX 100 chelating resin. The purified ^47^Sc was used to radiolabel 1,4,7,10-tetraazacyclododecane-1,4,7,10-tetraacetic acid-_D_Phe-c (Cys-Tyr-_D_Trp-Lys-Thr-Cys)-Thr-ol (DOTATOC) and the in vitro binding of [^47^Sc]Sc-DOTATOC was then established in somatostatin receptor subtype 2 (SSTR2)-expressing AR42J cells. These results show that future photonuclear production of ^47^Sc at a high-intensity eLINAC facility could represent a production route capable of producing quantities needed for pre-clinical research and translation to the clinic of this important theranostic radionuclide.

## Materials and methods

### General materials

All chemicals were analytical or trace metals grade and used as received unless otherwise noted. Milli-Q deionized 18.2 MΩ cm water (Milli-Q System; Millipore, Billerica, MA, USA) was used unless otherwise specified. Ammonium acetate (NH_4_CH_3_CO_2_, 99.999%), sodium hydroxide (NaOH, pellets, 99.995%), hydrogen peroxide (H_2_O_2_, TraceSELECT® Ultra ≥ 30%), hydrochloric acid (HCl, 37% wt. in water, 99.999%), nitric acid (HNO_3_, 70% wt. in water, 99.999%), and sulfuric acid (H_2_SO_4_, 95–98% wt. in water, 99.999%) were purchased from Sigma-Aldrich (St. Louis, Missouri, USA). Chelex® 100 chelating resin (100–200 mesh, Na^+^ form) and AG MP-50 cation exchange resin (100–200 mesh, H^+^ form) were purchased from Bio-Rad (Hercules, California, USA). 1,4,7,10-Tetraazacyclododecane-1,4,7,10-tetraacetic acid-_D_Phe-c (Cys-Tyr-_D_Trp-Lys-Thr-Cys)-Thr-ol (DOTATOC) and l-Cysteinamide, d-phenylalanyl-l-cysteinyl-l-phenylalanyl-d-tryptophyl-l-lysyl-l-threonyl-N-[(1R,2R)-2-hydroxy-1-(hydroxymethyl)propyl]-, cyclic (2 → 7)-disulfide (Octreotide) were purchased from Bachem Americas, Inc. (Torrance, CA, USA). Type 4 N high-purity, 0.127-mm-thick, natural titanium foil was purchased from ESPI Metals (Ashland, OR, USA). Scandium (III) nitrate hydrate (Sc (NO_3_)_3_, FW: 240.97 g/mol, 99.9%) was purchased from Alfa Aesar (Heysharn, Lancashire, UK). Stock solutions of 0.25 and 1.0 M ammonium acetate buffer, pH adjusted to 4.7, were purified with columns containing Chelex® 100 resin prior to use. All glassware was acid washed in a 50% HNO_3_ bath overnight at room temperature.

### Instrumentation

A modified Varian CLINAC (2100, Palo Alto, CA, USA) was used for all irradiations. The CLINAC was described in previous work by Queern et al. (2018) [25]. Similar to previous studies, a thin high Z material (tungsten) placed immediately upstream of the Ti target was used to enhance bremsstrahlung radiation. The average electron beam current was calibrated using an open-air AC current transformer fabricated by Bergoz Instrumentation (Saint Genis Pouilly, France). For calibration measurements, the transformer was mounted in the same location as the target holder. An electron beam energy of 22 MeV, the top setting with this CLINAC, was selected to maximize the photon flux in the region most likely to contain the highest photonuclear cross section for ^nat^Ti(γ,p)^47^Sc.

### Photonuclear production

The radionuclide ^47^Sc was produced via the ^48^Ti(γ,p)^47^Sc nuclear reaction by irradiation of a ^nat^Ti target foil stack (39 foils, 2.0 × 2.0 × 0.135 mm) using bremsstrahlung radiation generated by impinging 22 MeV electrons onto a 0.762-mm-thick tungsten radiator.

### Target dissolution

For each target foil, the region where the beam struck the foils was determined by imaging select foils for several hours with Gafchromic EBT3 radiographic film (Bridgewater, NJ, USA). The exposed films were used to create templates, which allowed for mechanical removal of activated Ti. The activated Ti was extracted from the target foils as discs using a portable lever-operated hole punch with a 3–4-mm punch (Douglasville, GA, USA). Activated discs were split into three sets and each set was treated similarly. Briefly, target foils were placed in a 50 mL round bottom flask along with 20 mL of 2.0 M H_2_SO_4_. The flask was fitted with a reflux condenser, and the apparatus was heated at 350 °C for 4 h using a sand filled mantle heater. During heating, the colorless solution became deep purple and remained clear. After heating, 1.16 mL of 30% H_2_O_2_ was added, resulting in a prompt color change from purple to intense orange. The solution was quantitatively transferred to a 100 mL beaker, whereupon 59 mL of water was added to adjust the H_2_SO_4_ molarity to 0.5 M.

### Resin conditioning

Prior to use, AG MP-50 and Chelex-100 resin were treated to remove contaminants. Briefly, 50 g of AG MP-50 resin was transferred to a clean 500 mL glass media bottle. First, the resin was mixed with 20 mL of 5 M HNO_3._ After settling, the supernatant was decanted to remove fine resin particles and contaminants. This was repeated twice. Next, the resin was mixed with 20 mL of water. After settling, the supernatant was decanted. This was repeated until the water, after mixing, had a pH of 7. The above process was then repeated using 2 M HCl. Finally, the treated resin was stored under water until use.

### Separation method

A separation method previously described by Kolsky et al. was adapted for this work [26]. A glass column (0.5 × 20 cm) was slurry packed with treated AG MP-50 cation exchange resin to a wet bed height of 2.5 cm. A glass wool plug was placed atop the resin bed. The resin was equilibrated by passing 15 mL of 0.5 M H_2_SO_4_ containing 2% H_2_O_2_ through the column at a flow rate of 1.5 mL/min. The digested target, containing ^47^Sc (in approximately 80 mL of 0.5 M H_2_SO_4_), was loaded onto the column at a flow rate of 1.5 mL/min. Adsorbed Ti was eluted with 40 mL of 2% H_2_O_2_ in 2.0 M H_2_SO_4_ at a flow rate of 1.5 mL/min. The resin was equilibrated with 10 mL of water and the adsorbed ^47^Sc was eluted with 20 mL of 1 M ammonium acetate buffer (pH 4–5). This eluate, containing ^47^Sc, was loaded onto a column (0.5 × 20 cm) containing Chelex® 100 chelating resin packed and prepared under conditions as described above. Adsorbed ^47^Sc was rinsed with 20 mL of water and eluted in 5.0 mL of 12.1 M HCl. After elution, the eluate was evaporated to a wet salt by gentle heating under nitrogen gas flow. This residue was dissolved in 1 mL of water and evaporated to dryness. This process was completed twice, resulting in a final product pH of 7. After the final evaporation the residue was dissolved in 400 μL of 0.25 M ammonium acetate (pH 4) which was used as the ^47^Sc solution for subsequent radiolabeling reactions. A typical separation and purification required 4 h.

### Elemental analysis

Fractions preserved from separations in this work were analyzed via inductively coupled plasma mass spectrometry (ICP-MS) to determine the concentration of elements present. Multi-element standards in 2% HNO_3_ at 1, 50, 100, 250, 500, 750, and 1000 mg/L were used for instrument calibration. For each collected fraction, an aliquot was diluted in 2% HNO_3_ and analyzed on an Agilent Technologies 7800 ICP-MS (Santa Clara, CA, USA) to determine the presence of transition metals. Agilent software, ICP-MS MassHunter v4.3, was used for data acquisition and analysis.

### γ-ray spectroscopy

Samples containing radionuclides in this work were characterized and quantified by γ-ray spectroscopy using a Canberra GC2018 high-purity germanium (HPGe) detector with a relative efficiency of 24.5% (Meriden, CT, USA) connected to a Canberra DSA-100 multichannel analyzer (Meriden, CT, USA). Canberra Genie 2000 software was used for all data acquisition and analysis (Meriden, CT, USA). An energy and efficiency calibration was performed using a mixed nuclide source in a sealed 1.5 mL microcentrifuge vial prepared by Eckert & Ziegler Analytics (Atlanta, GA, USA). Efficiency-calibration spectra with a minimum photopeak area of 2 × 10^5^ counts were collected and fit to a fourth-order polynomial to determine the detector efficiency [27]. For all measurements, aliquots of sample material were transferred to a 1.5 mL vial, diluted to 1 mL, and suspended in a fixed acrylic holder at a distance of 5 mm from the HPGe detector. This geometry was maintained for all samples. The activity of characterized radioisotopes was determined based on the net peak area (*N*_A_) in each photopeak [28]. For each photopeak, the activity (*A*_0_) at the beginning of the decay-counting measurement was determined according to the following equation:

$$ {A}_0=\frac{\uplambda {N}_{\mathrm{A}}}{I_{\upgamma}\upvarepsilon \left(1-\mathrm{DT}\right)\left(1-{\mathrm{e}}^{-\uplambda {t}_{\mathrm{R}}}\right)} $$

where λ is the decay constant of the radioisotope, *I*_γ_ is the branching ratio of the γ-ray, ε is the energy-dependent photopeak efficiency of the detector, DT is average dead time during the measurement, and *t*_R_ is the real time of measurement. For all measurements, the average dead time was less than 5%.

### Radiosynthesis of [^47^Sc]Sc-DOTATOC

As described by Pruszynski et al., radiolabeling of DOTATOC (1421.60 g/mol) with ^47^Sc was conducted in pH 4–5 0.25 M ammonium acetate buffer [29]. In brief, 60 μL of a 6.4 μg/μL aqueous DOTATOC stock solution was added to 40 μL of 0.25 M ammonium acetate buffer in a 1.5 mL centrifuge vial. To this vial, 1.7 MBq of ^47^Sc dissolved in 200 μL of 0.25 M ammonium acetate was added. The reaction pH and final concentration of DOTATOC (270 nmol in 300 μL) was pH 4–5 and 0.9 mM, respectively. The reaction mixture was heated for 30 min at 95 °C using an Eppendorf Thermomixer® C fitted with a 1.5 mL heating block at 800 rpm.

### [^47^Sc]Sc-DOTATOC characterization

Radiochemical analyses were performed using thin-layer chromatography (TLC) and high-performance liquid chromatography (HPLC). TLC analyses were performed using Agilent Technologies instant TLC-SA (iTLC) chromatography paper (Santa Clara, CA, USA) developed using a 1:1 mixture of 1.0 M ammonium acetate and methanol. TLC plates were read using an Eckert & Ziegler AR-2000 radioTLC system (Hopkington, MA, USA) and Eckert & Ziegler Winscan 3.0 software (Hopkington, MA, USA) was used to define regions of interest (ROI) and integrate peaks. HPLC analyses were performed on an Agilent Technologies HPLC (Infinity 1260, Santa Clara, CA, USA) equipped with a multi-wavelength diode-array detector followed by an in-line LabLogic detector (Flow-RAM Galbi NaI, Brandon, FL, USA) using a Thermo Scientific C18 analytical column (BetaBasic, 150 mm × 4.6 mm × 5 μm, 150 A, Waltham, MA, USA). LabLogic Laura™ radiochromatography software was used for HPLC data collection and analysis. Before any measurement by HPLC, solvents (HPLC grade) were degassed and water used for HPLC mobile phase was passed through a 0.22 μm filter. The HPLC method involved use of two solvents (A & B). Solvent A was acetonitrile (ACN) with 0.1% trifluoroacetic acid (TFA) and Solvent B was water with 0.1% TFA. The gradient elution began at 5%(A):95%(B) and changed linearly to 80%(A):20%(B) over 20 min at 1.0 mL/min.

### Cell culture

All cell culture materials were purchased from Fisher Scientific unless otherwise noted. The SSTR2-positive rat pancreatic cancer cell line AR42J (ATCC, Manassas, VA, USA) was cultured in Gibco™ IMDM media supplemented with 10% fetal bovine serum (FBS) and 0.1% gentamicin. Cells were incubated at 37 °C in a 5% CO_2_ atmosphere and were harvested using 0.05% trypsin-EDTA. Cells were washed once with supplemented IMDM prior to use. Cells were counted with a Nexcelom (Lawrence, MA, USA) auto T4 bright field cell counter.

### Competitive binding assay

AR42J cells were seeded into two 24-well plates (8 wells per plate) in 1 mL medium (1 × 10^5^ cells/well) and incubated overnight. After incubation, media was discarded, and 1 mL of 75 kBq [^47^Sc]Sc-DOTATOC (12 μM) in IMDM media was transferred into each non-blocking well (4 wells/plate). For the blocking study, 1 mL of 75 kBq [^47^Sc]Sc-DOTATOC containing 40 μM Octreotide was added into each blocking well (4 wells/plate). Additional 1 mL aliquots of the blocking and non-blocking solutions were transferred into empty wells on each plate as a reference. One milliliter of each solution, blocking and non-blocking, was transferred to separate 1.5 mL centrifuge tubes and used as reference standards (^47^Sc/1 mL dose) to determine total activity added to each well. Subsequently, the two 24-well plates were incubated at 37 °C for 2 h (first plate) or 4 h (second plate). At each time point, cells were washed three times with PBS, lysed with 1.0 M NaOH, and transferred into 1.5 mL centrifuge tubes. All centrifuge tubes were counted in a HIDEX 300 SL automatic γ-counter using a 3″ NaI crystal (Turku, Finland). Data is expressed as percentage of added dose bound (% Bound) with the standard error of the mean (SEM). A one-way ANOVA test was applied to each set of data (blocking and non-blocking) at 2 h and 4 h to determine *P* values and difference between each observed mean.

## Results

### ^47^Sc production and separation

An average ^47^Sc yield (*n* = 3) of 8 × 10^7^ Bq/mA h was obtained using a 22 MeV electron beam with an average beam current of 1.5 μA (Table 1).

**Table 1:** The parameters for each irradiation are shown

Run	EOB	Time (min)	Average current (μA)	Radiator THK (mm)	Titanium Foils (no.)
1	03/29/2017 17:03:00	630	2.5	0.762	39
2	05/07/2017 11:35:00	680	2.5	0.762	40
3	10/06/2017 20:51:00	840	2.5	0.762	39

**Fig. 2:**
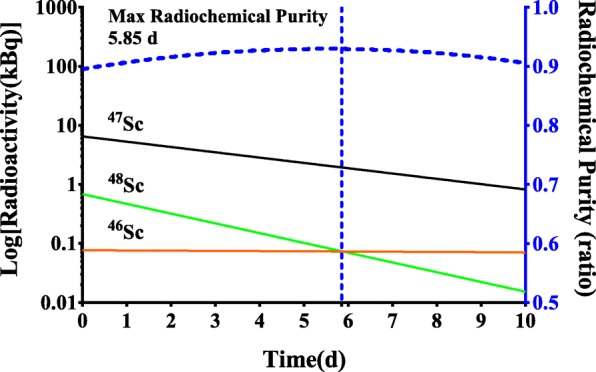
(Left axis) The radioactive decay of ^46,47,48^Sc and (right axis) ^47^Sc radioisotopic purity produced during the third irradiation modeled over 10 days

Using radiographic film, the region of produced ^47^Sc was mapped in select Ti foils. The beam strike, imaged on the film, of the entry and exit foil had an average diameter of 3 and 4 mm, respectively. Select foils were characterized and the presence of ^46^Sc (*T*_1/2_ = 83.79 days, 889.3 keV), ^47^Sc, and ^48^Sc (*T*_1/2_ = 1.82 days, 983.5, 1037.5, and 1312.1 keV) were confirmed by gamma ray analysis using characteristic gamma rays. The end-of-bombardment (EOB) radioisotopic purities of ^47^Sc, ^46^Sc, and ^48^Sc were determined by gamma ray analysis. The average EOB radioisotopic purity (*n* = 9) was (90.0 ± 0.1)% ^47^Sc, (1.2 ± 0.1)% ^46^Sc, and (8.3 ± 0.1)% ^48^Sc. Figure 2 shows the radioactive decay of ^46,47,48^Sc and ^47^Sc radioisotopic purity as a function of time for irradiation 3.

**Fig. 3:**
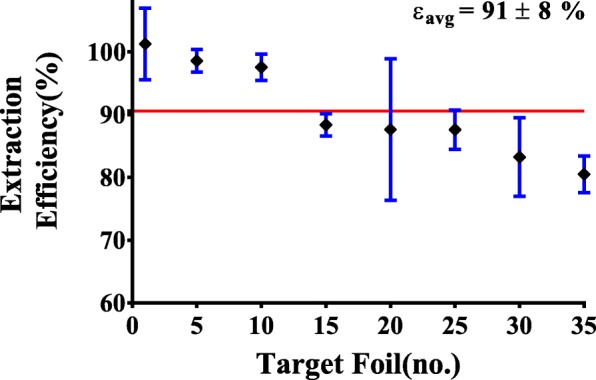
The average extraction efficiency (*n* = 3) expressed as the ratio of radioactivity measured in the extracted Ti disc versus the irradiated Ti foil. The interpolated average efficiency through the target foil stack (red trace). The beam was first incident upon target foil 1

The maximum radioisotopic purity was reached at approximately 5.85 days post-EOB (Fig. 2). Templates were fashioned using the exposed films and the region containing the greatest concentration of ^47^Sc was mechanically extracted from each ^nat^Ti target foil. The average extraction efficiency (*n* = 3) was (91 ± 8)% (Fig. 3).

For each irradiation, the extracted discs were split into three groups (A, B, and C) and dissolved in 2 M H_2_SO_4_ under reflux conditions. After dissolution, the solution was cooled to room temperature. The solution was clear and purple. A 1 mL aliquot was removed from each group and analyzed with an HPGe detector to measure the activity associated with each radionuclide. Table 2 shows the average decay-corrected EOB ^46,47,48^Sc radioactivity for each irradiation (sum of A, B, and C activities).

**Table 2:** The average (*n* = 3, 1σ) ^46,47,48^Sc radioactivity measured in ^nat^Ti discs processed in this work

Run	^46^Sc(kBq)	^47^Sc(kBq)	^48^Sc(kBq)
1	26 (1)	2178 (7)	229 (1)
2	25 (1)	2111 (124)	210 (17)
3	32 (1)	2420 (138)	222 (14)

In order to control Ti speciation, the digestion solution was adjusted to contain 2% of 30% H_2_O_2_ dissolved in 0.5 M H_2_SO_4_. Upon addition of hydrogen peroxide, the solution rapidly changed color to an intense orange but remained clear. In previous work, Muller and Schwarzenbach characterized the orange peroxytitanium (IV) species in solutions below pH 1, in general form, as Ti(O_2_)(OH)_n-2_^(4-n)+^; however, the number of hydroxyl groups has not been shown conclusively [30]. The ^46,47,48^Sc and peroxytitanium species mixture was directly loaded onto a column containing the treated AG MP-50 resin.

For the final optimized separation and purification (run 3), an aliquot was taken at each point in the separation and analyzed by HPGe and ICP-MS. Due to weak peroxytitanium affinity for AG MP-50 resin at low H_2_SO_4_ concentration, the absorbed ^nat^Ti was recovered in the flow-through and 1 M H_2_SO_4_ eluate. The recovery (*n* = 3) of bulk ^nat^Ti target material was quantitative as measured by ICP-MS. After the first eluent, the AG MP-50 resin was rinsed with 10 mL of water to clear hydrogen peroxide from the column. The strongly adsorbed ^46,47,48^Sc was then recovered in 20 mL 1 M ammonium acetate (pH 4.7). The average decay-corrected radioscandium recovery (*n* = 3, 1σ) was (92.2 ± 4.7)%. The concentration of metal contaminants, including Cr, Cu, Fe, Ni, and Zn, were measured in the 20 mL of the 1 M ammonium acetate eluate. The average concentration (*n* = 3, 1σ) of each trace metal contaminant was 191 ± 15 μg/L (Cr), 188 ± 36 μg/L (Cu), 414 ± 89 μg/L (Fe), 100 ± 30 μg/L (Ni), and 620 ± 296 μg/L (Zn) in 20 mL after this first column. A second column using CHELEX 100 was used to further purify the ^46,47,48^Sc solution. The ^46,47,48^Sc in ammonium acetate (pH 4.7) was directly loaded onto a column containing CHELEX 100 resin whereupon the ^46,47,48^Sc was strongly adsorbed. After rinsing with water, ^46,47,48^Sc was stripped from the column in 5 mL of 12.1 M HCl with an average (*n* = 3, 1σ) decay-corrected recovery of (91 ± 3)%. The total decay-corrected average (*n* = 3, 1σ) recovery was (83 ± 3)%. Similar to the above analysis, metal contaminants with a concentration > 1 μg/L were measured in the 5 mL of 12.1 M HCl eluate. The average concentration (*n* = 3, 1σ) of Cr, Cu, Fe, Ni, and Zn was 127 ± 6 μg/L (Cr), 169 ± 34 μg/L (Cu), 357 ± 154 μg/L (Fe), 107 ± 33 μg/L (Ni), and 189 ± 25 μg/L (Zn) in 5 mL. These data are shown in Table 3.

**Table 3:** The average mass (*n* = 3, 1σ) of contaminant metals measured in the digestant and the eluate recovered from the AG MP-50 and CHELEX 100 resin

Sample	Cr	Cu	Fe	Ni	Zn
Digestant mass (μg)	552 (30)	493 (72)	946 (35)	206 (24)	908 (620)
Post AG MP-50 (μg)	3.9 (0.3)	3.8 (0.7)	8.4 (1.8)	2.0 (0.6)	13 (6)
Post CHELEX 100 (μg)	0.6 (0.2)	0.8 (0.2)	1.8 (0.8)	0.5 (0.2)	1.9 (0.1)

The overall reduction in Cr, Cu, Fe, Ni, and Zn after separation and purification was 99.9%, 99.8%, 99.8%, 99.8%, and 99.9%, respectively.

**Fig. 4:**
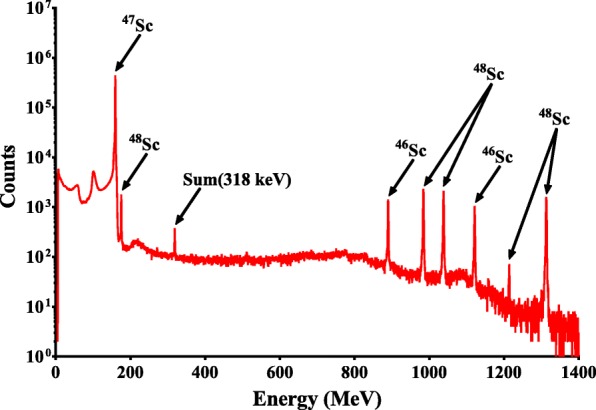
HPGe spectra of the purified radiolabeling solution. The photopeaks attributed to ^46,47,48^Sc have been characterized and labeled. The sum peak due to the intense ^47^Sc photopeak at 159 keV has been noted

For the final irradiation (run 3), the 12.1 M HCl eluates containing ^46,47,48^Sc(III) from groups A, B, and C were combined and evaporated to a wet residue under nitrogen gas with gentle heating (95 °C). The residue was dissolved in 1 mL of water and evaporated. This process was repeated twice more. After the final evaporation was conducted, the residue was dissolved in 255 μL of 0.25 M ammonium acetate (pH 4) after which a 10 μL aliquot was taken and analyzed. Figure 4 shows the characterized HPGe spectra of the final product.

This solution contained a ^47^Sc concentration of 8.5 ± 0.4 kBq/μL. The average concentration of trace metal contaminants in 255 μL of solution were 0.8 ± 0.1 mg/L (Cr), 0.8 ± 0.1 mg/L (Cu), 1.6 ± 0.1 mg/L (Fe), 1.8 ± 0.1 mg/L (Ni), and 6.0 ± 0.3 mg/L (Zn).

### Radiosynthesis of [^47^Sc]Sc-DOTATOC

To radiolabel DOTATOC, 200 μL of 0.25 M ammonium acetate (pH 4.7) containing 1.7 MBq of ^47^Sc was added to 100 μL of 0.1 M ammonium acetate (pH 4) containing 0.384 mg of DOTATOC (270 nmol). The final concentration of DOTATOC was 0.9 mM with a molar activity of 6.3 kBq/nmol. The mixture was vortexed and incubated at 95 °C for 30 min.

For TLC analyses, in a 1:1 mixture of 1.0 M ammonium acetate and methanol, free ^47^Sc remained at the origin (*R*_f_ = 0). The TLC analysis of a 1 μL aliquot from the [^47^Sc]Sc-DOTATOC solution showed a single peak with an *R*_f_ value of 0.8. For HPLC analyses, the retention times of free ^47^Sc and DOTATOC were 1.9 min and 8.8 min, respectively. The HPLC analysis of the [^47^Sc]Sc-DOTATOC solution diluted with 0.25 M ammonium acetate showed a peak in the radio and UV/Vis (λ = 280 nm) spectra at 8.5 min and 8.8 min, respectively. These analyses showed the radiochemical yield was > 99.9% and thus the compound was used without further purification. The HPLC and TLC chromatograms of the [^47^Sc]Sc-DOTATOC solution are shown in the supplemental data (Additional file 1: Figure S1).

### Competitive cell binding assay

**Fig. 5:**
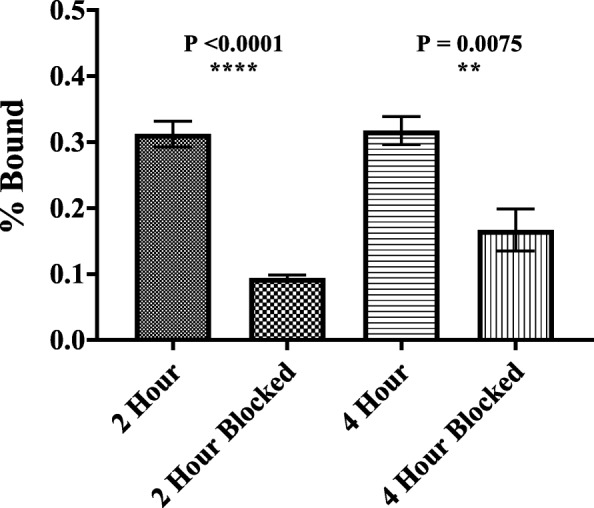
The percentage of receptor bound or internalized [^47^Sc]Sc-DOTATOC incubated with AR42J cells is shown on the ordinate axis at 2 and 4 h (*n* = 4, 1σ, SEM). Co-incubation with SSTR blocking agent Octreotide significantly decreased cell uptake

Binding of [^47^Sc]Sc-DOTATOC in AR42J cells was determined in vitro at 2 and 4 h. To determine if uptake of [^47^Sc]Sc-DOTATOC was receptor-mediated, the ligand Octreotide was used as a blocking agent due to its known affinity for SSTR2 [31, 32]. At both 2 and 4 h (*n* = 4), the percentage of [^47^Sc]Sc-DOTATOC bound to cells was significantly decreased (*p* < 0.0001 and *p* = 0.0075) in cells that were co-incubated with Octreotide (Fig. 5).

The maximum mean uptake and standard error of [^47^Sc]Sc-DOTATOC was achieved by 2 h (0.31 ± 0.02)%, as uptake at 4 h (0.32 ± 0.02)% was not significantly increased.

## Discussion

The multiple irradiations conducted with the modified Varian CLINAC allowed for measurements of the target foil activities and beam dispersion throughout a ^nat^Ti target foil stack. The diameter of the activated region increased from 3 to 4 mm throughout 39 ^nat^Ti foils. For each foil, the region containing ^47^Sc was mechanically extracted, reducing the bulk quantity of Ti involved in the separation. While this method and the use of natural titanium metal was suitable for this work, future studies that seek to optimize ^47^Sc yield will require enriched titanium (IV) oxide (^48^Ti) targets, well-defined target geometry, target cooling, and optimized target mass due to the strong dependence between yield and the target nuclei population. Further, the use of an oxide target with a profile commensurate to the bremsstrahlung beam diameter will not require mechanical extraction to reduce unirradiated target material or high temperature methods for the reductive synthesis of Ti (0), required to recover target material after an aqueous chemical separation. Importantly, as the target mass increases, the selected separation method must be able to sequester gram quantities of target material, which must be recovered and recycled, for iterative irradiations. As future work continues on developing photonuclear production of ^47^Sc, these parameters should be optimized for high-intensity electron beams as they will have a direct impact on recovery and purity of the chemically isolated ^47^Sc.

A number of scandium radionuclides may be co-produced when using a ^nat^Ti target, including ^44^Sc, ^46^Sc, ^48^Sc, and ^49^Sc. Similar to the photonuclear work previously reported by Mamtimin et al., ^46^Sc (*T*_1/2_ = 83.79 days) and ^48^Sc (*T*_1/2_ = 43.7 h) were experimentally observed in the Ti foils; however, ^44^Sc and ^49^Sc were not observed due to their short half-lives and maximum bremsstrahlung photon energy used in this work [10]. The co-production of ^46^Sc presents the most significant challenge to using ^nat^Ti as a target material since the ^47^Sc radionuclidic purity decreases over time due to the longer half-life of ^46^Sc. The two contaminant radionuclides can, in principle, be produced via the ^48^Ti(γ,np)^46^Sc, ^47^Ti(γ,p)^46^Sc, ^50^Ti(γ,np)^48^Sc, and ^49^Ti(γ,p)^48^Sc reactions and have threshold energies of 22.1 MeV, 10.5 MeV, 22.3 MeV, and 11.3 MeV, respectively.

In the work by Mamtimin, using a 22 MeV electron beam, the EOB ratio of ^46,47,48^Sc activities were 0.03, 0.82, and 0.14, respectively [10]. In the work by Rotsch et al., using a 35 MeV electron beam and capturing a larger portion of the ^nat^Ti(γ,p)^47^Sc cross section (Fig. 1), the ratio of ^46,47,48^Sc EOB activities produced were 0.01, 0.90, and 0.09 [20]. The ratio of ^46,47,48^Sc EOB activities in this work using a 22 MeV electron beam were 0.01, 0.90, and 0.09. For each study, ^nat^Ti target material was used and the irradiation times were less than one half-life of the three radionuclides. These studies show that the shape of the excitation function for each radionuclide was similar, resulting in comparable radionuclidic purity at EOB even at different electron beam energies. Furthermore, when using a 22 MeV electron beam in our work, less than the threshold energies of the (γ,np) channels, no improvement in ^47^Sc radionuclidic purity was observed when compared to the work by Mamtimin and Rotsch, suggesting the (γ,p) reactions on ^47^Ti and ^49^Ti are the dominant sources of Sc impurities.

The ^47^Sc EOB radionuclidic purity was impacted by co-production of ^46^Sc and ^48^Sc for each study when using ^nat^Ti target material. Thus, the best case scenario for limiting co-production of contaminants, ^46^Sc and ^48^Sc, involves the use of enriched ^48^Ti target material, available commercially at enrichment levels up to 99.6% [33]. Favorably, the abundant ^48^Ti (73.8%) may be purchased commercially at a relatively low cost (2–3$/mg) [33].

The extracted Ti foils were acid digested and a separation method using AG MP-50 cation exchange resin and CHELEX 100 resin was used to isolate the ^47^Sc. This separation system was selected based on previously published work using both resins [26, 34]. Initial elemental analysis of fractions preserved during separation with AG MP-50 showed varying levels of trace metal contamination due to Cr, Cu, Fe, Ni, and Zn. To reduce the concentration of these cations, which may compete with radioscandium for DOTA binding or cause transmetallation of the radioscandium-DOTA complex, a second separation step was performed to further purify the isolated ^47^Sc [35]. For this two-step method, the overall recovery of ^47^Sc for the separation and purification of was (77 ± 7)%. The observed losses during radiochemical separation were due to material transfer and loss to both the AG MP-50 and CHELEX 100 resins. The typical masses of impurities measured after both steps were less than 1 μg for Cr, Cu, and Ni while Fe and Zn was less than 2 μg. These data on overall recovery compare well with recent work by Rotsch et al. (2018) and Domnanich et al. (2017) where a 76.3% and 89.7% overall recovery was reported using branched DGA (*N*,*N*,*N*′,*N*′-tetrakis-2-ethylhexyl-diglycolamide) cation-exchange resin [20, 36]. Further, the mass of Zn and Fe measured in the purified ^47^Sc fraction was reduced by 60% and 45%, respectively, compared to the mass reported by Rotsch et al. (2018) [20].

As a proof of concept, the purified ^47^Sc was used to radiolabel DOTATOC, a somatostatin analog that when radiolabeled can be used for diagnosis and therapy of neuroendocrine tumors that overexpress SSTR2. This small peptide was selected due to its well-characterized affinity for trivalent radiometals [37]. A high radiochemical yield of 99.9% was achieved for the radiosynthesis of [^47^Sc]Sc-DOTATOC, which was subsequently used in an in vitro cell uptake assay in SSTR2-expressing AR42J cells. The maximum uptake was achieved by 2 h, and co-incubation of the radiolabeled compound with Octreotide lead to a 70% reduction in uptake at 2 h, indicating receptor-mediated uptake. In previous work, [^43,44^Sc]Sc-DOTATOC has been synthesized and evaluated for SSTR2-binding; however, the work performed in this study illustrates the feasibility of performing initial radiosynthetic and pre-clinical screening studies using the moderate ^47^Sc yields obtained from a CLINAC [13, 29, 38, 39].

## Conclusion

This work demonstrated the photonuclear production of ^47^Sc using a modified Varian CLINAC, tungsten radiator, and targets composed of ^nat^Ti foil. These results show that CLINACs no longer in clinical use may be used for the production of limited quantities of medically relevant radionuclides for small-scale experiments. Moreover, the methods shown in this work when applied to increased ^47^Sc production yields would likely result in higher molar activities. Based on this work, future photonuclear production of ^47^Sc using enriched ^48^Ti targets and high-intensity eLINACs may be a feasible route to developing this promising theranostic radionuclide; however, the limited cross-section data on the ^48^Ti(γ,p)^47^Sc reaction must be improved in order to optimize radionuclidic purity and ^47^Sc yield.

## Additional file

Additional file 1: **Figure S1.** (A) HPLC of DOTATOC (blue, t_R_ = 8.8 min) and radio-HPLC of ^47^Sc (red, t_R_ = 1.9 min). (B) HPLC of DOTATOC (blue, t_R_ = 8.8 min) and radio-HPLC of [^47^Sc]Sc-DOTATOC (red, t_R_ = 8.5 min). (C) Radio-TLC of ^47^Sc (R_f_ = 0) and (D) [^47^Sc]Sc-DOTATOC (Rf = 0.8) in 0.25 M ammonium acetate developed in a 1:1 mixture of 1.0 M ammonium acetate and methanol. (PDF 55 kb)

## Abbreviations

eLINAC: Electron linear accelerator
HPGe: High purity germanium
HPLC: High-pressure liquid chromatography
PET: Positron emission tomography
ROI: Region of interest
SEM: Standard error of the mean
SSTR: Somatostatin receptor subtype 2
TLC: Thin-layer chromatography

## References

[1] Srivastava SC, Mausner LF, Baum RP (2014). Therapeutic radionuclides: production, physical characteristics, and applications. Therapeutic nuclear medicine.

[2] Jeong H-J, Lee BC, Ahn B-C, Kang KW (2016). Development of drugs and Technology for Radiation Theragnosis. Nucl Eng Technol.

[3] Baum RP, Kulkarni HR (2012). THERANOSTICS: from molecular imaging using Ga-68 labeled tracers and PET/CT to personalized radionuclide therapy - the Bad Berka experience. Theranostics..

[4] Nicolas GP, Mansi R, McDougall L, Kaufmann J, Bouterfa H, Wild D (2017). Biodistribution, pharmacokinetics, and dosimetry of (177) Lu-, (90) Y-, and (111) in-labeled somatostatin receptor antagonist OPS201 in comparison to the agonist (177) Lu-DOTATATE: the mass effect. J Nuclear Med.

[5] Gudkov SV, Shilyagina NY, Vodeneev VA, Zvyagin AV (2016). Targeted radionuclide therapy of human tumors. Int J Mol Sci.

[6] Rösch F, Herzog H, Qaim MS. The beginning and development of the theranostic approach in nuclear medicine, as exemplified by the radionuclide pair 86Y and 90Y. Pharmaceuticals. 2017;10(2):56. 10.3390/ph10020056 PMC5490413 28632200

[7] Srivastava SC (2012). Paving the way to personalized medicine: production of some promising Theragnostic radionuclides at Brookhaven National Laboratory. Semin Nucl Med.

[8] Leveque DW, Sandra JF (2005). Pharmacokinetics of therapeutic monoclonal antibodies used in oncology. Anticancer Res.

[9] Fosgerau K, Hoffmann T (2015). Peptide therapeutics: current status and future directions. Drug Discov Today.

[10] Mamtimin M, Harmon F, Starovoitova VN (2015). Sc-47 production from titanium targets using electron linacs. Appl Radiat Isot.

[11] Frank R (2012). Scandium-44: benefits of a long-lived PET radionuclide available from the 44Ti/44Sc generator system. Curr Radiopharm.

[12] Bartoś B, Majkowska A, Krajewski S, Bilewicz A. New separation method of no-carrier-added 47Sc from titanium targets. Radiochimica Acta International Journal For Chemical Aspects Of Nuclear Science and Technology2012. p. 457.

[13] Müller C, Bunka M, Haller S, Köster U, Groehn V, Bernhardt P (2014). Promising prospects for 44Sc−/47Sc-based theragnostics: application of 47Sc for radionuclide tumor therapy in mice. J Nucl Med.

[14] Misiak R, Walczak R, Wąs B, Bartyzel M, Mietelski JW, Bilewicz A (2017). 47Sc production development by cyclotron irradiation of 48Ca. J Radioanal Nucl Chem.

[15] Gadioli E, Gadioli Erba E, Hogan JJ, Burns KI (1981). Emission of alpha particles in the interaction of 10–85 MeV protons with48,50Ti. Zeitschrift für Physik A Atoms Nuclei.

[16] DeLorme K, Engle J, Kowash B, Nortier F, Birnbaum E, McHale S (2014). Production potential of Sc-47 using spallation neutrons at the Los Alamos isotope production facility. J Nucl Med Meeting Abstracts.

[17] Habs D, Köster U (2011). Production of medical radioisotopes with high specific activity in photonuclear reactions with γ-beams of high intensity and large brilliance. Applied Physics B.

[18] Belyshev SS, Dzhilavyan LZ, Ishkhanov BS, Kapitonov IM, Kuznetsov AA, Orlin VN (2015). Photonuclear reactions on titanium isotopes. Phys At Nucl.

[19] Starovoitova VN, Cole PL, Grimm TL (2015). Accelerator-based photoproduction of promising beta-emitters 67Cu and 47Sc. J Radioanal Nucl Chem.

[20] Rotsch DA, Brown MA, Nolen JA, Brossard T, Henning WF, Chemerisov SD (2018). Electron linear accelerator production and purification of scandium-47 from titanium dioxide targets. Appl Radiat Isot.

[21] Sherwood TR, Turchinetz WE (1962). Some photo-disintegration reactions in the titanium isotopes. Nucl Phys.

[22] Bodnar EN, Dikiy MP, Medvedeva EP (2015). Photonuclear production and antitumor effect of radioactive cisplatin (195mPt). J Radioanal Nucl Chem.

[23] Smith NA, Bowers DL, Ehst DA (2012). The production, separation, and use of 67Cu for radioimmunotherapy: a review. Appl Radiat Isot.

[24] Grimm TL, Boulware CH, Hollister JL, Jecks RW, Mamtimin M, Starovoitova V. Commercial superconducting electron linac for radioisotope production. ; Niowave, Inc., Lansing, MI (United States); 2015. Report No.: 13–0019--FTR-0001; Other: 13–0019 United States 10.2172/1209691 Other: 13-0019 CHO English.

[25] Queern SL, Cardman R, Loveless CS, Shepherd MR, Lapi SE. Production of 15O for Medical Applications via the 16O(gamma,n)15O Reaction. J Nucl Med. 2019;60:424-8. 10.2967/jnumed.118.215681 30237213

[26] Kolsky KL, Joshi V, Mausner LF, Srivastava SC (1998). Radiochemical purification of no-carrier-added scandium-47 for radioimmunotherapy. Appl Radiat Isot.

[27] Gray PW, Ahmad A (1985). Linear classes of Ge (li) detector efficiency functions. Nucl Inst Methods Phys Res A.

[28] Wooten AL, Lewis BC, Lapi SE (2015). Cross-sections for (p,x) reactions on natural chromium for the production of (52,52m,54) Mn radioisotopes. Applied radiation and isotopes : including data, instrumentation and methods for use in agriculture, industry and medicine.

[29] Pruszyński M, Majkowska-Pilip A, Loktionova NS, Eppard E, Roesch F (2012). Radiolabeling of DOTATOC with the long-lived positron emitter 44Sc. Appl Radiat Isot.

[30] Schwarzenbach G, Muehlebach J, Mueller K (1970). Peroxo complexes of titanium. Inorg Chem.

[31] Anthony L, Freda PU (2009). From somatostatin to octreotide LAR: evolution of a somatostatin analogue. Curr Med Res Opin.

[32] Patel YC, Srikant CB (1994). Subtype selectivity of peptide analogs for all five cloned human somatostatin receptors (hsstr 1-5). Endocrinology..

[33] Luu A (2018). Ti isotopes budgetary pricing.

[34] Strelow FWE. Distribution coefficients and ion exchange behavior of 46 elements with a macroreticular cation exchange resin in hydrochloric acid 1984. 10.1016/0039-9140(88)80032-8 18964538

[35] Oehlke E, Le V, Lengkeek N, Pellegrini P, Jackson T, Greguric I (2013). Influence of metal ions on the Ga-68-labeling of DOTATATE.

[36] Domnanich KA, Eichler R, Müller C, Jordi S, Yakusheva V, Braccini S (2017). Production and separation of 43Sc for radiopharmaceutical purposes. EJNMMI Radiopharmacy and Chem.

[37] Liu S (2004). The role of coordination chemistry in the development of target-specific radiopharmaceuticals. Chem Soc Rev.

[38] Hernandez R, Valdovinos HF, Yang Y, Chakravarty R, Hong H, Barnhart TE (2014). 44Sc: an attractive isotope for peptide-based PET imaging. Mol Pharm.

[39] Walczak R, Krajewski S, Szkliniarz K, Sitarz M, Abbas K, Choiński J (2015). Cyclotron production of (43) Sc for PET imaging. EJNMMI Physics.

