# Supplementary figures and images for: Photonuclear production, chemistry, and in vitro evaluation of the theranostic radionuclide 47Sc
Source: EJNMMI Res. 2019 May 16;9:42. doi: 10.1186/s13550-019-0515-8 (PMC6522578; doi:10.1186/s13550-019-0515-8)

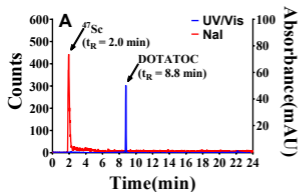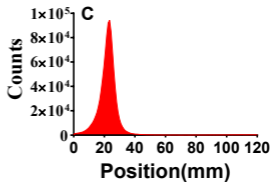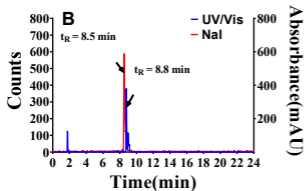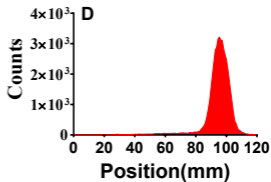

Supplement: Supplementary file 1 — Figure S1. (A) HPLC of DOTATOC (blue, tR = 8.8 min) and radio-HPLC of 47Sc (red, tR = 1.9 min). (B) HPLC of DOTATOC (blue, tR = 8.8 min) and radio-HPLC of [47Sc]Sc-DOTATOC (red, tR = 8.5 min). (C) Radio-TLC of 47Sc (Rf = 0) and (D) [47Sc]Sc-DOTATOC (Rf = 0.8) in 0.25 M ammonium acetate developed in a 1:1 mixture of 1.0 M ammonium acetate and methanol. (PDF 55 kb) [file 13550_2019_515_MOESM1_ESM.pdf]
